# Beyond contextual stressors: uncertain reflective functioning, loneliness and emotional exhaustion as psychological correlates of perceived stress in prison officers

**DOI:** 10.1108/IJOPH-09-2025-0074

**Published:** 2026-01-27

**Authors:** Antonia Sorge, Alessandro Musetti, Gianluca Bianchi, Mattia Pezzi, Christian Franceschini, Denise Vagnini, Emanuela Saita

**Affiliations:** Department of Psychology, Università Cattolica del Sacro Cuore, Milan, Italy; Department of Humanities, Social Sciences and Cultural Industries, University of Parma, Parma, Italy; Department of Psychology, Università Cattolica del Sacro Cuore, Milan, Italy; Department of Humanities, Social Sciences and Cultural Industries, University of Parma, Parma, Italy; Department of Medicine and Surgery, University of Parma, Parma, Italy; Department of Psychology, Università Cattolica del Sacro Cuore, Milan, Italy

**Keywords:** Stress, Reflective functioning, Loneliness, Emotional exhaustion, Prison officers, Correctional officers, Job stress

## Abstract

**Purpose:**

This study aims to examine how uncertain reflective functioning (RF_U), loneliness (isolation, relational connectedness and trait loneliness) and emotional exhaustion (EE) contribute to perceived stress among prison officers (POs), beyond traditional contextual factors.

**Design/methodology/approach:**

A cross-sectional study was conducted with a sample of 58 Italian POs (mean age = 44.3 years; 72.4% male, 27.6% female) who completed standardized self-report measures distributed via Qualtrics. Descriptive statistics, Pearson correlations and multiple regression analyses were conducted to examine associations among variables.

**Findings:**

Correlation analysis indicated that higher perceived stress was linked to greater loneliness, particularly low relational connectedness, higher EE and greater RF_U. However, only relational connectedness and RF_U significantly predicted perceived stress in the multiple regression analysis, controlling for age, gender and job type, highlighting their key role in shaping stress responses.

**Originality/value:**

These findings suggest the potential utility of interventions targeting both external conditions and internal relational processes to effectively reduce perceived stress in POs.

## Introduction

The work environment of prison officers (POs) can be challenging, as they encounter persistent occupational, organizational and situational stressors that may contribute to elevated work-related stress and burnout (Bezerra *et al.*, 2016; Clements *et al.*, 2020; Jaegers *et al.*, 2020). Several research studies have linked POs’ stress to factors such as heavy workloads, role ambiguity, poor interpersonal relationships and exposure to critical incidents (Beijersbergen *et al.*, 2015; Costa *et al.*, 2024; Farnese *et al.*, 2017; Kilmer *et al.*, 2023; Kinman *et al.*, 2017; Schaufeli and Peeters, 2000; Schiff and Leip, 2019). Institutional conditions – including overcrowding, rigid hierarchies, insufficient organizational support or recognition, public criticism, legal accountability and poor environmental conditions (e.g. inadequate lighting, poor air quality, high noise levels, sanitation issues and limited nature contact) – may further intensify stress (Bernheimer, 2017; Engstrom and van Ginneken, 2022; Law and Guo, 2016; Ricciardelli *et al.*, 2024; Sorge *et al.*, 2023).

Contextual stressors are well documented, but little is known about the psychological mechanisms behind POs’ stress perception. While most studies have focused on demographics such as age and gender, meta-analyses show these variables have a minor impact (Bottaro *et al.*, 2023; Dowden and Tellier, 2004; Miller *et al.*, 2007). Exploring psychological dimensions could improve understanding of stress among POs.

Mentalization theory (Fonagy *et al.*, 2016) provides a useful framework in this context. Mentalization, defined operationally as reflective functioning (RF) – the ability to understand and interpret one’s own and others’ behavior in terms of mental states (Fonagy, 1989; Fonagy *et al.*, 2003) – is associated with how individuals, including those within non-clinical populations, regulate stress (Schwarzer *et al.*, 2022). RF typically develops through early caregiver interactions, where parental responsiveness and mentalizing support enhance the child’s capacity to understand both their own and others’ thoughts and emotions. Conversely, insecure or disorganized attachment patterns may hinder the development of RF, potentially increasing vulnerability to disturbances in self-concept, emotional regulation and the onset of psychopathology (Fonagy and Target, 1997; Fonagy *et al.*, 2003). Traumatic experiences, whether occurring in early childhood or later in life, may impair RF (Fonagy *et al.*, 2003; Fonagy *et al.*, 2023), possibly leading individuals to perceive themselves as lacking control over life events and increasing susceptibility to psychological distress (Barazzone *et al.*, 2019; Cannavò *et al.*, 2025). Within the mentalizing process, two dysfunctional patterns have been identified: uncertain RF (RF_U, or hypomentalizing) and certain RF (or hypermentalizing). RF_U involves a concrete, rigid and inflexible approach to interpreting behavior, which limits the ability to recognize the complexity and ambiguity of mental states – both their own and those of others. This is commonly referred to as uncertainty about mental states. In contrast, certain RF involves the tendency to over-attribute mental states without sufficient evidence or regard for objective reality. Both aspects of impaired mentalizing are associated with difficulties in emotional regulation, interpersonal functioning and maladaptive coping strategies (Luyten *et al.*, 2020).

Recent studies underscore the relevance of RF in high-stress jobs. Zhou *et al.* (2023) and Wang *et al.* (2024) found that elevated RF_U is associated with increased stress perception among health-care workers. Similarly, Manzano-García *et al.* (2021) revealed that entrepreneurs with high RF_U are more prone to burnout, particularly manifesting as cynicism and negative interpersonal attitudes toward others. To date, only one study has explored mentalizing processes among Law Enforcement Officers (LEOs), suggesting a distinctive profile characterized by prioritization of others over self, reliance on external cues, predominance of cognitive over affective processing, implicit mentalizing under stress and avoidance of vulnerable emotional states (Drozek *et al.*, 2021). According to this work, LEOs tend to reflexively focus on others’ behavior and experiences, while finding introspection and self-reflection less intuitive. This mentalizing profile confers occupation-specific advantages, such as the ability to adaptively “disconnect” from emotions (e.g. fear) during critical incidents. However, these same patterns of mentalizing may also pose risks, including empathic deficits in interpersonal conflict (Drozek *et al.*, 2021; Valdespino *et al.*, 2017). Furthermore, impaired mentalizing capacities can foster negative social expectations and withdrawal from relationships, ultimately increasing vulnerability to isolation (Luyten and Fonagy, 2015). Recent evidence also links impaired mentalizing to heightened loneliness (Pouravari *et al.*, 2023), underscoring its potential role as a psychosocial risk factor in law enforcement populations.

Loneliness may play an important role in perceived stress among POs. Defined as a distressing experience resulting from the discrepancy between desired and actual social relationships (Peplau and Perlman, 1982), Weiss (1973) distinguished loneliness into emotional (i.e. a lack of a close and intimate bond with others) and social (i.e. a lack of a social group or community to which the person belongs). This distinction has been further refined by recent evidence (Boffo *et al.*, 2012; Bottaro *et al.*, 2023), which identified three distinct dimensions of loneliness: isolation, relational connectedness and trait loneliness. Isolation refers to the subjective sense of being socially excluded or detached from others; relational connectedness reflects the perceived lack of emotionally significant relationships; trait loneliness represents trait-like factors (e.g. shyness, low self-confidence) that increase vulnerability to loneliness across different life contexts (Boffo *et al.*, 2012; Boffo *et al.*, 2012; Bottaro *et al.*, 2023). Empirical evidence indicates that loneliness has been associated with cognitive biases, coping abilities and biological stress responses, including dysregulation of the hypothalamic–pituitary–adrenal axis (Cacioppo and Hawkley, 2009). Laustsen *et al.* (2024) found a bidirectional relationship between loneliness and perceived stress, but this connection has not yet been examined in POs. This gap is relevant given evidence that supportive workplace relationships can mitigate psychological distress in this population (Ricciardelli *et al.*, 2021).

In addition to RF_U and loneliness, emotional exhaustion (EE) is a central construct in understanding stress, particularly within occupational contexts. EE is the feeling of depletion caused by prolonged job stress (Bryan *et al.*, 2023; Fernet *et al.*, 2016; Seppala and King, 2017) that is associated with an individual’s reduced ability to remain affectively engaged with their work and social environment (Maslach *et al.*, 2001). Among POs, EE is particularly prevalent because of continuous exposure to emotionally demanding situations such as workplace aggression and high occupational demands (Bottaro *et al.*, 2023; Clements and Kinman, 2022). This condition has been consistently associated with negative outcomes, including lower life satisfaction (Ricciardelli *et al.*, 2021) and poorer psychological well-being (Bernheimer, 2017). Comparable associations have also been observed in other human service professions, such as social workers (Chaves-Montero *et al.*, 2025). Prison work has been conceptualized as “emotional labor” (Miller *et al.*, 2007), encompassing relational and affective demands that extend beyond formal procedural tasks required to maintain order and security (Crawley, 2013; Forsyth *et al.*, 2025; Corpo di Polizia Penitenziaria, 2025). Core duties may constitute “emotional dirty work” (McMurray and Ward, 2014), reflecting the stigma and emotional burden inherent in the role. To manage these demands, POs may engage in emotional distancing and identity-protection strategies (Dick, 2005; Ricciardelli *et al.*, 2021), which, while functional in the short term, may weaken interpersonal bonds and contribute to loneliness.

Taken together, these theoretical and empirical foundations highlight the importance of exploring psychological vulnerabilities in understanding perceived stress among POs. The present study examines the associations between RF_U, loneliness and EE with perceived stress in POs. Specifically, controlling demographic variables, we propose the following hypothesis:

*H1*. Higher RF_U will be associated with higher perceived stress

*H2*. Higher loneliness will be associated with higher perceived stress

*H3*. Higher EE will be associated with higher perceived stress

By investigating these associations, the study aims to provide preliminary evidence regarding psychological mechanisms that may be related to stress in this high-risk professional group.

## Method

### Procedure

The study was conducted in accordance with the Declaration of Helsinki and received approval from the Ethics Commission of *** (protocol number ***). Data were collected anonymously using a convenience sampling method. Participants voluntarily accessed the online questionnaires via a QR code provided by the research team during the two weeks preceding the start of a psychological support intervention implemented across three correctional institutions at different times throughout 2023 and 2024. The QR code directed participants to a Qualtrics-hosted survey, where they were presented with an electronic informed consent form. Only individuals who provided informed consent could proceed. Participation in the survey was entirely voluntary. Because the survey was anonymous, participants could withdraw their consent at any time before submitting the questionnaire by closing the browser window. In such cases, no data were recorded or used for the study. Once the questionnaire was submitted, data could no longer be withdrawn because of the absence of identifying information. To reduce the likelihood of duplicate submissions, the Qualtrics platform was configured to allow only one response per device or IP address.

### Participants

An *a priori* power analysis was conducted using G*Power version 3.1.9.6 (Faul *et al.*, 2007) to determine the minimum sample size required to test the study hypotheses. For correlational analyses, the power analysis indicated that a minimum sample size of 49 participants was required to detect a correlation of *r = *0.35 with 80% power at a 5% significance level (two-tailed test), yielding an actual power of 0.80. For the first hierarchical multiple regression analysis, the power analysis determined that a minimum of 59 participants was necessary to detect an effect size of *f^2^* = 0.20 with 80% power at a 5% significance level. This hierarchical model with two steps examined the associations between multiple predictor variables and the dependent variable. Three predictors were introduced in the second step, with a total of six predictors included across both steps of the regression model. Similarly, for the second hierarchical multiple regression analysis, the power analysis indicated that 58 participants were required to detect an effect size of *f^2^* = 0.25 with 80% power at a 5% significance level. This model included five predictors added in the second step, with a total of eight predictors across both steps.

Participants were required to be active members of the Italian prison police force. A total of 71 participants completed the survey. However, 14 respondents were excluded during data cleaning because they did not identify as POs. These individuals were identified based on an initial multiple-choice question regarding their professional role within the correctional facility. The final sample comprised 58 Italian POs employed in three correctional institutions within the same northern Italian region. Average overcrowding was 185%, while the proportion of foreign inmates was 49.5% (Ministero della Giustizia, 2024). Participation rates relative to available staff were approximately 20% in two institutions and 17% in the third. Participants had a mean age of 44.3 years (SD =* *9.91; range: 20–57) and an average of 19.1 years of service (SD =* *11.8; range: 1–36). Gender distribution was 72.4% male and 27.6% female. Most officers originate from regions different from their assigned facility but typically settle permanently near the institution; only younger officers (early twenties) tend to reside temporarily in barracks before relocating locally. Given the sample’s mean age, it is unlikely that participants belonged to this transitional group. because of institutional constraints, data on living arrangements or proximity to family were unavailable.

Regarding job roles, 60.3% (*n* =* *35) of respondents held administrative positions, while 39.7% (*n* =* *23) worked directly in prison sections supervising and managing incarcerated persons. Most participants were operational staff in the *Agenti* and *Assistenti* ranks. A small number of *Ispettori* (two participants) were also included because of their supervisory duties overlapping with frontline staff. Higher managerial ranks were not represented.

Prison work involves planned role rotation approximately every two years, encompassing administrative tasks, perimeter surveillance, accounting, inmate transfers, escort duties, internal support services (e.g. bar) and residential-unit supervision. Administrative and support functions are numerically more prevalent than residential-unit duties, where staffing is typically limited to one or two officers per floor (about four to five officers per unit).

### Demographic data

Participants provided demographic information such as age, gender, years of service and job type. Job type was categorized as administrative role for those in office-based duties and custodial role for those working directly within prison sections. These variables described the sample and were used to control for associations with stress perception and psychological measures.

## Measures

### Perceived stress scale

The Perceived Stress Scale (PSS-10; Cohen and Williamson, 1988; Italian version by Fossati, 2010) is a ten-item self-report questionnaire designed to evaluate how individuals perceive stress in their lives. It measures the cognitive and emotional experience of stress over the past month. Items (e.g. “In the last month, how often have you felt nervous and ‘stressed’?”) are rated on a five-point Likert scale ranging from 0 (*never*) to 4 (*very often*). The scale includes four reverse-scored items (Items 4, 5, 7 and 8), and the total score ranges from 0 to 40, with higher scores indicating greater levels of perceived stress. The psychometric properties of the Italian scale were evaluated by Mondo *et al.* (2021), who found that the PSS-10 outperformed the 14- and 4-item versions in terms of reliability and validity. More recently, Messineo and Tosto (2024) confirmed the PSS-10’s robustness in an Italian sample of teachers, supporting its use across diverse populations. In the current study, PSS-10 demonstrated good internal consistency (*α* = 0.815). Although the PSS-10 does not specifically assess occupational stress, it was considered appropriate for evaluating perceived stress in POs, as it has been successfully used in previous research involving correctional officers (Bezerra *et al.*, 2016; Owen, 2006).

### Reflective functioning questionnaire

The Reflective Functioning Questionnaire (RFQ-8; Fonagy *et al.*, 2016; Italian version by Morandotti *et al.*, 2018) was used to assess difficulties in mentalizing. The RFQ-8 comprises two subscales, each containing six items that measure the degree of uncertainty (RFQ_U) and certainty (RFQ_C) regarding mental states. The two subscales are moderately and negatively correlated (*r =* –0.403, *p* <* *0.05), as they share four out of six items. For the purposes of this study, only the Uncertainty subscale (RFQ_U) was used to assess levels of uncertain RF. This subscale consists of six items rated on a seven-point Likert scale ranging from *completely disagree* (1) to *completely agree* (7). An example item is: “Sometimes I do things without really knowing why.” Following Morandotti *et al.* (2018), responses are recoded as 0, 0, 0, 0, 1, 2 and 3, such that higher scores reflect greater uncertainty about mental states. The Italian version of the RFQ-8 has demonstrated good reliability and validity. In the present study, the RFQ_U subscale showed good internal consistency (Cronbach’s *α* = 0.865).

### UCLA Loneliness Scale – Version 3

The UCLA Loneliness Scale – Version 3 (UCLA LS3; Russel, 1996; Italian version by Boffo *et al.*, 2012) is a self-report questionnaire designed to measure feelings of loneliness through 20 items rated on a four-point Likert scale, ranging from *never* (1) to *always* (4). An example item of UCLA LS3 is: “How often do you feel alone?” In the current study we used a three-factor structure of the scale (Boffo *et al.*, 2012), considering three dimensions: isolation; relational connectedness; and trait loneliness. This structure was recently confirmed by Bottaro *et al.* (2023), who also reported strong internal consistency (Cronbach’s *α* = 0.96; Russell, 1996) and good test–retest reliability (*r = *0.73). The scale includes nine reverse-scored items (1, 5, 6, 9, 10, 15, 16, 19, 20), with total scores ranging from 20 to 80. Higher UCLA LS3 scores indicate greater loneliness. In the present study, internal consistency for the total scale was high (Cronbach’s *α* = 0.894). Cronbach’s alpha coefficients were 0.765 for isolation, 0.861 for relational connectedness and 0.523 for trait loneliness.

### Maslach Burnout Inventory

The Maslach Burnout Inventory (MBI; Maslach and Jackson, 1986; Italian version by Sirigatti and Stefanile, 1992) is a self-report measure designed to assess burnout syndrome, characterized by EE, depersonalization and reduced personal accomplishment. For this study, we focused exclusively on the EE subscale, which assesses work-related fatigue and emotional depletion. The EE subscale includes nine items (e.g. “I feel emotionally exhausted because of my work”), rated on a seven-point Likert scale ranging from *never* (0) to *every day* (6). Higher scores indicated greater EE. The Italian version of the MBI demonstrated adequate psychometric properties (Sirigatti and Stefanile, 1992), making it a reliable tool for assessing burnout in various helping professions. To confirm internal consistency, we computed Cronbach’s alpha, which indicated good internal reliability (*α* = 0.871).

## Data analysis

All statistical analyses were conducted using Jamovi (Version 2.5) [Computer Software]. The data set included responses from 58 participants. Both descriptive and inferential statistical analyses were performed to examine the relationships among the study variables. Descriptive statistics, including mean, standard deviation, minimum and maximum values, skewness and kurtosis, were computed for all measures. To assess associations among the variables, a correlation matrix was computed. Given that RF_U exhibited substantial non-normality and a floor effect because of a high frequency of zero values, the Spearman rank-order correlation coefficient was used for correlation involving RF_U. All other correlations were computed using Pearson’s correlation coefficient. Pearson correlations were calculated to evaluate associations between perceived stress, loneliness and its dimensions, EE, gender, age, years of service and job type. Spearman correlations were used to assess associations between RF_U and the same set of variables.

A hierarchical multiple regression analysis was performed using the ordinary least squares estimation method to examine the extent to which socio-demographic and psychological factors were associated with perceived stress. Assumptions for conducting hierarchical regression, including the evaluation of multicollinearity, were assessed. The analysis aimed to assess the unique and combined statistical associations of the predictor variables while controlling for demographic factors. The predictors included socio-demographic variables (age, gender and job type), RF_U, the dimensions of loneliness (i.e. isolation; relational connectedness; and trait loneliness) and EE. Although both age and years of service were collected as sociodemographic variables, only age was included in the regression model because of a high correlation between the two variables (*r = *0.696). Including both would have introduced multicollinearity, potentially compromising the stability of the regression estimates. This regression analysis was designed to explore the associational patterns between perceived stress and socio-demographic, psychological and loneliness-related factors. It is important to note that the results are correlational in nature and do not imply causal relationships.

Although post hoc power analyses have well-known conceptual limitations (Zhang *et al.*, 2019), they can offer useful descriptive information when interpreting non-significant findings (Onwuegbuzie and Leech, 2004). For this reason, we conducted a post-hoc power analysis using G*Power 3.1 (Faul *et al.*, 2007). Assuming a medium effect size (*f^2^* = 0.15), *α* = 0.05, eight predictors and *n* =* *58, the achieved power was 0.82. This level of power suggests that the study had adequate sensitivity to detect medium-sized associations under the specified analytical conditions.

## Results

Descriptive statistics, including means, standard deviations, minimum and maximum values, skewness and kurtosis, were computed for all key study variables (see Table 1).

**Table 1:** Descriptive statistics for key study variables (*n* = 58)

Measure	*M* (SD)	Observed range	Skewness	Kurtosis
Age	44.3 (9.91)	20–57	−0.812	−0.617
Years of service	19.1 (11.75)	1–36	−0.398	−1.379
Emotional exhaustion	14.0 (11.60)	1–45	1.08	0.427
Uncertain reflective functioning	0.282 (0.359)	0–1.88	1.993	5.765
Perceived stress	23.8 (5.60)	12–43	0.591	1.23
Loneliness total score	40.9 (8.71)	21–70	0.535	0.972
Isolation	7.91 (2.45)	4–14	0.466	−0.619
Relational connectedness	25.9 (5.70)	13–45	0.671	1.21
Trait loneliness	7.17 (1.81)	4–11	0.213	−0.517

Note(s):

M = mean; SD = standard deviation

**Source(s):** Authors’ own work

Pearson’s correlations (see Table 2) showed that perceived stress was significantly associated with greater EE (*r = *0.290, *p* <* *0.05) and total loneliness (*r = *0.621, *p* <* *0.001), especially with the relational connectedness subscale (*r = *0.638, *p* <* *0.001). Spearman’s rank-order correlation showed that perceived stress was also significantly associated with RF_U (*ρ* = 0.512; *p* <* *0.001).

**Table 2:** Correlation analysis (*n* = 58)

Variable		Age	Years of service	Gender	Job type	Perceived stress	Loneliness total score	Isolation	Relational connectedness	Trait loneliness	Emotional exhaustion	Uncertain reflective functioning
Age	*r*	—										
	*p*-Value	—										
Years of service	*r*	0.696***	—									
	*p*-Value	<0.001	—									
Gender	*r*	−0.518***	−0.345*	—		−0.154	−0.095	−0.137	−0.074	−0.038	−0.114	
	*p*-Value	<0.001	0.011	—		0.249	0.480	0.306	0.583	0.778	0.397	
Job type (POs_admin; POs_custody)	*r*	−0.578***	−0.463***	0.288*	—	−0.193	−0.285*	−0.218	−0.230	−0.353**	−0.046	
	*p*-Value	<0.001	<0.001	0.028	—	0.146	0.030	0.101	0.083	0.007	0.732	
Perceived stress	*r*	0.219	0.131	−0.154	−0.193	—						
	*p*-Value	0.098	0.346	0.249	0.146	—						
Loneliness total score	*r*	0.163	0.091	−0.095	−0.285*	0.621***	—	0.820***	0.960***	0.675***		
	*p*-Value	0.223	0.515	0.480	0.030	<0.001	—	<0.001	<0.001	<0.001		
Isolation	*r*	0.102	0.000	−0.137	−0.218	0.474***	0.820***	—				
	*p*-Value	0.448	1.000	0.306	0.101	<0.001	<0.001	—				
Relational connectedness	*r*	0.146	0.071	−0.074	−0.230	0.638***	0.960***	0.689***	—			
	*p*-Value	0.273	0.611	0.583	0.083	<0.001	<0.001	<0.001	—			
Trait loneliness	*r*	0.183	0.216	−0.038	−0.353**	0.332*	0.675***	0.419**	0.533***	—		
	*p*-Value	0.169	0.117	0.778	0.007	0.011	<0.001	0.001	<0.001	—		
Emotional exhaustion	*r*	0.027	0.119	−0.114	−0.046	0.290*	0.354**	0.380**	0.296*	0.255	—	
	*p*-Value	0.844	0.398	0.397	0.732	0.028	0.007	0.004	0.025	0.056	—	
Uncertain reflective functioning	*ρ*	0.193	0.090	−0.346**	−0.031	0.512***	0.341**	0.232	0.391**	0.147	0.056	—
	*p*-Value	0.148	0.517	0.008	0.820	<0.001	0.009	0.080	0.002	0.271	0.678	—

Note(s):

*p* <* *0.05 (*), *p* <* *0.01 (**), *p* <* *0.001 (***)

**Source(s):** Authors’ own work

EE was moderately correlated with total loneliness (*r = *0.354, *p* <* *0.01), especially with the isolation subscale (*r = *0.380, *p* <* *0.01), but was not significantly associated with RF_U (*ρ* = 0.056, *p* =* *0.678).

RF_U was positively correlated with loneliness, particularly with the relational connectedness subscale (*ρ* = 0.391, *p* <* *0.01), and negatively correlated with gender (*ρ* = −0.346, *p* <* *0.01), indicating that male POs were more likely to report higher RF_U.

Regarding socio-demographic variables, job type was negatively correlated with both age and years of service (see Table 2), suggesting that administrative roles were more frequently held by older POs, while younger officers were more often assigned to custody roles. Job type was also negatively correlated with loneliness (*r =* −0.285; *p* <* *0.05), specifically with trait loneliness, indicating that administrative POs reported lower levels of trait loneliness (*r =* −0.353; *p* <* *0.01).

A hierarchical multiple regression analysis was conducted to examine associations between socio-demographic and psychological factors and perceived stress. Socio-demographic variables (age, gender and job type) were entered in Step 1. In Step 2, psychological factors, including RF_U, the dimensions of loneliness (isolation, relational connectedness and trait loneliness) and EE, were added to assess their incremental contribution to the model. In Step 1, socio-demographic variables accounted for a non-significant proportion of variance in perceived stress [*R^2^* = 0.047, adjusted *R*^2^ = 0.012, *F*(2,54) = 1.35, *p* =* *0.268]. In Step 2, the addition of psychological variables significantly improved the model fit, explaining 53.25% of the variance in perceived stress [*R^2^* = 0.532, adjusted *R^2^* = 0.454, *F*(8,48) = 6.83, *p* <* *0.001]. A model comparison test confirmed that the inclusion of psychological factors significantly increased the explained [Δ*R^2^* = 0.485, *F*(6, 48) = 8.30, *p* <* *0.001].

Regression coefficients for the final model are reported in Table 3.

**Table 3:** Hierarchical regression predicting perceived stress

Predictor	Estimate	*β*	*t*	95% CILL − UL	p	VIF	Tolerance
Intercept	6.320	–	1.249	–	0.218	–	–
Age	0.045	0.080	0.568	−0.203 to 0.363	0.573	2.04	0.491
Gender (F–M)	0.759	0.134	0.502	−0.404 to 0.673	0.618	1.52	0.660
Job type (POs_custody vs POs_admin)	−0.201	−0.035	−0.132	−0.577 to 0.507	0.895	1.80	0.555
Emotional exhaustion	0.064	0.132	1.220	−0.085 to 0.351	0.573	1.21	0.827
Uncertainty reflective functioning	5.457	0.349	3.068	0.120 to 0.579	0.004**	1.33	0.750
Isolation	0.167	0.072	0.506	−0.216 to 0.362	0.615	2.13	0.470
Relational connectedness	0.418	0.426	2.684	0.107 to 0.745	0.010**	2.59	0.387
Trait loneliness	0.097	0.031	0.250	−0.220 to 0.283	0.804	1.61	0.621

Note(s):

*p* <* *0.01 (**)

**Source(s):** Authors’ own work

In the final model, relational connectedness was the variable most strongly related to perceived stress (*B *=* *0.418; *β* = 0.426; *p* =* *0.010), followed by RF_U (*B *=* *5.457; *β* = 0.349; *p* =* *0.004). In contrast, EE, socio-demographic variables and the other loneliness dimensions (isolation and trait loneliness) were not significantly associated with perceived stress (all *p*-values > 0.05).

## Discussion

This study examined if psychological factors – specifically RF_U, loneliness and EE – were associated with perceived stress in POs beyond demographic characteristics. The findings partly supported the hypotheses and offer insight into stress-related processes in this occupational group.

Among all variables considered, relational connectedness, reflecting dissatisfaction with close relationships, was identified as the strongest variable associated with perceived stress. This suggests that the quality of interpersonal bonds may be related to how stress is experienced among POs. Previous qualitative research has found that POs often describe emotional withdrawal and interpersonal disconnection from loved ones because of work-related strain, time demands and behavioral spillover from work into private life, leading to a gradual deterioration of social and emotional ties (McKendy and Ricciardelli, 2021). Family members have similarly reported experiencing emotional changes in POs, who may appear increasingly withdrawn, suspicious, or emotionally distant over time. Such patterns have been shown to spill over into family life, generating tension, distress and perception that aspects of prison routines are brought into the home environment (Crawley, 2002). These findings align with previous studies showing that work–family conflict is a significant source of stress among POs (Armstrong *et al.*, 2015).

Our results align with broader evidence indicating that relational connectedness – which reflects emotional loneliness – was identified as the strongest psychological correlate of perceived stress among POs. Notably, this study uses a multidimensional perspective on loneliness, which is increasingly regarded as crucial in both research and clinical contexts. Walsh *et al.* (2025) note that emotional and social loneliness are distinct constructs with different impacts on psychological well-being. Their review indicates that emotional loneliness is often more strongly associated with distress than social loneliness.

Relational difficulties may further intensify perceived stress. Previous research studies (Carter, 1996; Hua-Fu, 2005) have noted that prison environments often promote emotional suppression and stoicism. These behaviors discourage emotional expression and interpersonal openness, which may be associated with increased feelings of loneliness and with challenges in mentalizing. As De Viggiani (2012) observed, these behaviors can manage vulnerability and maintain professional legitimacy, yet they may conflict with fundamental human needs for connection and emotional support.

In line with our hypotheses, RF_U was positively associated with perceived stress. This finding aligns with mentalization theory (Fonagy *et al.*, 2003), which suggests that difficulties in understanding and interpreting one’s own and others’ mental states are linked to emotional dysregulation and maladaptive coping strategies. Recent research in high-stress occupational groups, including health-care and security professionals, has also highlighted the role of RF_U in increasing stress vulnerability (Safiye *et al.*, 2023; Schwarzer *et al.*, 2022; Zhou *et al.*, 2023; Wang *et al.*, 2024). When individuals have reduced access to mentalizing capacities, they may struggle to understand others’ behaviors, leading to interpersonal misunderstandings and increased psychological strain.

Within correctional contexts, these challenges may be intensified. The inability to accurately interpret the intentions or emotions of others may correspond to more rigid, black-and-white appraisals of social situations, increasing stress and undermining effective relational engagement. Occupational norms emphasizing emotional control and self-reliance may reinforce hypomentalizing tendencies. As D'angelo *et al.* (2018) found, POs often report low perceived competence in relational domains such as empathy and emotional support – skills that are important in rehabilitative prison models. This gap may result in emotional withdrawal and RF_U, particularly in environments where institutional culture limits opportunities for introspection and emotional connection. In our sample, we observed a significant negative correlation between gender (1 = male, 2 = female) and RF_U (*ρ* = −346; *p* <* *0.01), indicating that male officers reported higher levels of RF_U compared to female officers. While this result should be interpreted cautiously, it may suggest that male POs experience more difficulties engaging in reflective processes, a possibility that warrants further investigation in future studies.

In contrast to our hypothesis, EE, isolation and trait loneliness did not significantly predict perceived stress in the regression model despite moderate bivariate correlations. This may reflect shared variance with more proximal predictors such as relational connectedness and RF_U. The lack of a unique contribution from EE is particularly noteworthy, given its well-documented association with occupational stress in prison settings (Bottaro *et al.*, 2023; Clements and Kinman, 2022). One possible interpretation is that EE may represent a more distal or downstream correlate of prolonged stress exposure, whereas constructs such as RF and relational connectedness could represent more proximal and dynamic aspects of how stress is appraised and regulated. This interpretation is based on Fonagy *et al.* (2025), who suggest that the perception and evaluation of potentially stressful stimuli involve higher order cognitive functions – such as executive control, attention and self-awareness – which shape the individual’s emotional response. From this perspective, RF may act as a key psychological factor that facilitates adaptive processing of stress, while EE may represent a more cumulative outcome of repeated maladaptive appraisals over time. Nonetheless, the observed correlations between EE and loneliness – particularly the isolation dimension – remain theoretically meaningful. These findings resonate with research by Coppola *et al.* (2023), which showed that POs with limited relational competence and intercultural sensitivity reported higher EE and lower life satisfaction. Overall, this evidence highlights the importance of relational and emotional capacities not only in mitigating burnout symptoms but also in supporting more adaptive responses to occupational stress in demanding prison environments.

These findings offer preliminary insights into potential intervention targets. For example, training programs enhancing relational skills and emotional literacy could encourage more adaptive interpersonal functioning among POs. Furthermore, psychological support addressing loneliness and difficulties in mentalizing could be incorporated into staff well-being initiatives. Organizational strategies should also consider structural efforts to promote peer support and relational connectedness within correctional settings. These intervention targets would be consistent with emerging evidence from the broader literature. A recent systematic review (Woodall, 2024) highlighted the significant absence of high-quality research on health promotion interventions for prison staff, although some promising initiatives have been reported. For example, Smith *et al.* (2022) found that a brief yoga and mindfulness-based program improved coping abilities, emotional regulation and self-awareness among prison staff. While limited in scope, these findings support the relevance of interventions aimed at enhancing psychological resources in prison settings. However, the interpretations drawn from the present study should be considered preliminary and require replication. Further research is needed to confirm our findings in larger and more diverse samples before firm conclusions can be drawn about intervention strategies.

## Practical implications

Although preliminary, the present findings offer several implications for professional practice and organizational planning in correctional settings.

First, the associations observed between RF, relational connectedness and perceived stress suggest that training programs aimed at enhancing perspective-taking, emotional awareness and interpersonal understanding could be beneficial for POs. Mentalization-informed approaches or structured stress-management modules may help officers navigate the complex interpersonal demands inherent in their work.

Second, the relevance of relational connectedness underscores the importance of strengthening social support within correctional environments. Initiatives such as peer-support networks, structured team debriefings and mentoring programs can foster a more cohesive and emotionally supportive climate. Organizational practices that facilitate communication and reduce isolation – such as supervisory dialogues or access to psychological consultation – could further buffer stress in high-demand units.

Third, systematic monitoring of organizational climate and workload distribution can help identify units where targeted interventions are most needed. Enhancing relational and emotional competencies may not only mitigate stress but also contribute to safer and more constructive interactions within correctional settings.

Finally, these implications extend beyond correctional contexts. Similar dynamics may be relevant in policing and other high-stress occupational groups where emotional regulation, interpersonal engagement and social connectedness are critical. Future research should examine how interventions targeting these psychological dimensions operate across first responder and security professions.

## Limits and future directions

This study has several limitations. First and foremost, the cross-sectional design precludes any inference of causality. Although significant associations were found, their directionality cannot be determined. Longitudinal or experimental studies are needed to clarify temporal relationships and potential mediating pathways. Second, the sample size was relatively small and restricted to POs from one geographic region in Italy. Although adequate for the planned analyses, this limits the generalizability of the findings to broader national or international populations. Additionally, although operational differences between frontline and administrative roles may be relevant, the sample did not allow reliable subgroup analyses. Future research with larger samples could examine whether different job roles are associated with distinct psychological profiles or stress experiences.

With regard to procedures, although measures were taken to prevent duplicate responses – such as restricting Qualtrics to one submission per device or IP address – this strategy reduces but does not eliminate the risk of multiple entries by the same participant. Replication with larger, multi-site samples is therefore needed. Third, despite all measures used having been validated, data collection relied exclusively on self-report instruments, which may be susceptible to response biases such as social desirability or limited introspective accuracy – especially in assessing constructs such as mentalizing. Future research should consider incorporating behavioral assessments or multi-informant reports to enhance measurement validity. Another limitation concerns the internal consistency of the Trait Loneliness subscale, which was low (*α* = 0.523) compared with previous Italian validation studies (Boffo *et al.*, 2012; Bottaro *et al.*, 2023). This discrepancy may reflect sample-specific factors. In highly regulated institutional contexts such as prisons, traits associated with vulnerability (e.g. shyness, low self-confidence) may be underreported or misrepresented because of prevailing cultural norms that reward emotional control, independence and toughness. As a result, the scale may not have captured the construct reliably in this population. Future studies should further examine the psychometric performance of this subscale in professional contexts and consider adaptation or revalidation when applied to occupational samples.

Beyond addressing these limitations, future research should also examine potential mediating and moderating variables – such as attachment style, organizational climate, coping strategies or relational competencies – to better understand the mechanisms linking psychological vulnerabilities to stress in prison environments. Intervention studies assessing programs aimed at strengthening RF and promoting emotionally supportive work climates may offer valuable insights for stress prevention and the promotion of mental health among POs.

Overall, future studies would benefit from explicitly addressing the methodological limitations of the present work by using longitudinal designs, adopting multi-site recruitment strategies and increasing sample size. These improvements would enhance the robustness, generalizability and temporal interpretability of the associations identified in this study.

## Conclusion

This study extends existing research on perceived stress among POs by highlighting the relevance of RF and relational connectedness – two psychological dimensions that have been relatively underexplored in prison research. While socio-demographic variables were not significantly associated with stress, difficulties in mentalizing (supporting *H1*) and lower relational connectedness (supporting *H2*) were meaningfully associated with perceived stress.

EE showed moderate bivariate associations with stress but did not emerge as a unique predictor in the regression model, offering only partial support for *H3*.

These findings have potential implications for staff well-being and stress management initiatives within prison environments. Interventions aimed at enhancing RF and fostering emotionally supportive professional relationships may help mitigate perceived stress and promote psychological well-being among prison staff. Overall, the results support a broader and more integrative model of occupational stress – one that includes both contextual stressors and the internal psychological resources and vulnerabilities of individuals working in high-risk institutional contexts. By integrating RF and relational processes into models of occupational stress, this study contributes to a more comprehensive framework for understanding the emotional and interpersonal challenges faced by POs and highlights potential targets for future interventions.
